# Applying molecular genetic data at different scales to support conservation assessment of European Habitats Directive listed species: A case study of Eurasian otter in Austria

**DOI:** 10.1111/eva.13597

**Published:** 2023-09-27

**Authors:** Tamara Schenekar, Andreas Weiss, Steven J. Weiss

**Affiliations:** ^1^ Institute of Biology University of Graz Graz Austria; ^2^ NASA Postdoctoral Program Fellow NASA Goddard Space Flight Center Greenbelt Maryland USA; ^3^ Institute of Physics University of Graz Graz Austria

**Keywords:** biogeographic regions, genetic structure, *Lutra lutra*, microsatellites, otter densities

## Abstract

Evaluating intraspecific genetic structure and diversity is fundamental to assessing a species’ conservation status, but direct incorporation of such information into legal frameworks such as the EU's Habitats Directive is surprisingly rare. How genetic structure aligns with EU member state boundaries or biogeographic regions may be very important in designing management plans or achieving legislative goals. The Eurasian fish otter experienced a sharp population decline during the 20th century but is currently re‐expanding in several countries. The species is listed under Annex II and IV of the European Habitats Directive, and member states are obliged to assess the species separately across different biogeographic regions. We genotyped 2492 otter spraints across four provinces in Austria, collected between 2017 and 2021. A total of 384 different genotypes were identified, supporting densities along river habitats from 0.1 to 0.47 otters per river km (mean: 0.306), with a resampling‐based simulation supporting limited density overestimation at survey lengths of 20 km or more. Three distinct genetic clusters were revealed, two of them presumably reflecting two relict populations whereas the source of the third cluster is unknown. The geographic extent of the three clusters does not coincide with provincial or biogeographic boundaries, both relevant for assessment and management within existing national or European legislative frameworks. We advocate more consideration of genetic structure in the assessment and conservation management planning of species listed in the European Habitats Directive.

## 1 INTRODUCTION

### 1.1 Genetic approaches for conservation biology and wildlife management

Over the last two decades, molecular genetic tools have become valuable to fields of applied ecology and conservation biology and support efforts to (1) estimate census or effective population sizes (Luikart et al., 2010), (2) track individuals to, for example, infer home ranges (Taberlet et al., 1997), (3) identify predators from wildlife kills (Harms et al., 2015) or assess potential intraspecific structure to identify management or conservation units (Hohenlohe et al., 2021; Scribner et al., 2005; Segelbacher et al., 2010). Deriving management units from patterns of genetic structure has been done for numerous endangered or heavily harvested plants (Cohen & Ruane, 2023; Hartvig et al., 2020), fishes (Fisch et al., 2011; Hallerman & Hilsdorf, 2014; Vilas et al., 2010), amphibians (Forester et al., 2022; Krug et al., 2022), reptiles (Carlson et al., 2016; Ciofi et al., 1999; Jensen et al., 2014), birds (Eo et al., 2010; Renfrew et al., 2022) and mammals (Bilgin, 2012; Degner et al., 2007; Gonzalez et al., 2015; Tournayre et al., 2019). Spatial genetic data can also be highly informative for local or international conservation and management measures (Palsbøll et al., 2007; Schwartz et al., 2007). For example, the description of genetic diversity can identify risks of enhanced genetic drift due to small population sizes, which can lead to local inbreeding effects (Keller & Waller, 2002) and assessment of population differentiation can avoid outbreeding depression, which might hamper the efficiency of relocation efforts (Edmands, 2007), in cases when there are high levels of genetic divergence among populations. On a smaller scale, genetic information can be used to infer home ranges of individuals or dispersal behavior (Davoli et al., 2013; Paquette et al., 2010) and this information can be utilized in turn to design or evaluate the required minimum spatial extent of monitoring surveys for endangered or managed species. Despite the popularity of these approaches in a conservation context, the application of such information has not been universal, in part, due to differences in legislative or policy frameworks.

The United States’ Endangered Species Act (ESA, 1973,16 U.S.C. §§1531–1544) regularly incorporates genetic data to describe sub‐specific units of endangered or threatened species, particularly in defining distinct population units (DPS) (Kelly, 2010). For example, recently four out of six DPSs of the foothill yellow‐legged frog *Rana boylii* were proposed to be listed in the ESA (two as threatened and two as endangered) based on genomic data. In contrast, implementation of genetic data into conservation or management decisions related to the EU Habitats Directive (Council Directive 92/43/EEC) has been minimal. According to Frankham (2010) and Koskela et al. (2013), the lack of genetic‐based management in biodiversity conservation has not been due to the lack of research or guidelines, but due to failure to incorporate genetic aspects into practical management. Keller et al. (2015) give recommendations on how landscape genetic studies can be better integrated into conservation management, to, for example, optimize statistical species distribution models and align study areas with spatial conservation management units.

To date, geographic management units of the EU Habitats Directive are shaped by administrative borders (i.e., national borders of member states or provincial borders within member states) or biogeographic regions (regions within Europe defined by the European Environment Agency based on presumed faunal or floral community similarities European Environment Agency, 2017). However, whether these units coincide or not with genetic population structure is rarely evaluated.

In Austria, as in many other EU member states, the management of wildlife populations is conducted on the provincial level. These municipalities are required to assess the conservation status of each species listed under the European Habitats Directive (Council Directive 92/43/EEC). These assessments must be done separately for each biogeographic region, and within Austria, this relates to the alpine and continental biogeographic regions. Therefore, it would be relevant to evaluate to what extent genetic structure aligns with administrative or biogeographic units, especially if management across these units is not uniform.

### 1.2 Demographic history of the Eurasian fish otter in Europe and Austria

The Eurasian fish otter (*L. lutra*, Linnaeus 1758, hereafter otter) is listed in Annex II and IV of the European Habitats Directive since 1992. It was widespread throughout Europe during the 19th century but experienced a sharp population decline during the 20th century (Macdonald & Mason, 1994; Roos et al., 2015). During the early 1990s, the otter was reportedly absent in multiple central European countries. Relatively widespread populations were still reported from the Iberian Peninsula (Portugal and western Spain) and western to central France (Foster‐Turley et al., 1990). In Italy, a few small populations, most notably in Basilicata and Campania (Buglione et al., 2021) remained. From Eastern Europe, declines have been reported from Poland, Czech Republic, parts of Hungary and Bulgaria (Hájková et al., 2009; Lanszki et al., 2008; Romanowski et al., 2013).

**FIGURE 1:**
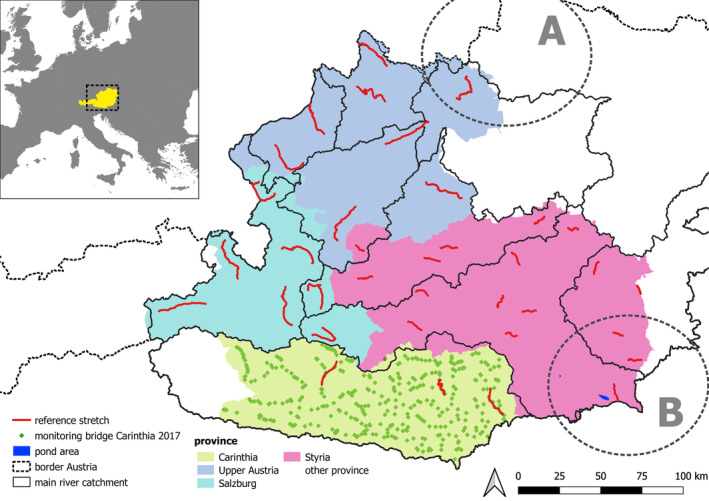
Sampled reference stretches and sites of the five surveys from four different Austrian provinces synthesized in this study. Green diamonds represent predefined monitoring bridges of the Carinthia 2017 survey. Red lines represent reference stretches along which samples were collected for the other four surveys. Blue polygons represent the two pond areas sampled in the Styrian survey. Colored polygons in the background represent the four Austrian provinces for which data are available. Black lines indicate borders of main river catchments in the study area. Dashed black line indicates border of Austria and dashed grey ellipses (A & B) indicate the approximate position of the two remnant otter populations during the 20th century. Inset shows the relative position of Austria on the European mainland.

In Austria, the otter remained present throughout the 20th century in two distinct refuge areas. One was in the North of the country in the provinces of Upper and Lower Austria, bordering the Czech Republic (Figure 1, ellipse a) and the second was in the South‐East of Austria in the provinces of Styria and Burgenland bordering Slovenia and Hungary (Figure 1, ellipse b).

Since the 1990s (Kruuk, 2006; Reuther, 1994), continuously expanding otter populations have been reported from central European countries (Buglione et al., 2021; Macdonald & Mason, 1994; Mucci et al., 2010), likely as a result of various protection measures. Similarly, otters were presumed to have expanded their distribution from the two described refuge areas in Austria (e.g., Kofler et al., 2018; Kranz & Poledník, 2009, 2013; Kranz et al., 2005). Currently, the otter shows an almost exhaustive distribution in the central and eastern parts of Austria, whereas West‐Austria (provinces Tyrol and Vorarlberg) is only partially inhabited (Schenekar et al., 2022).

The otter's drastic population decline and refugial isolation followed by re‐expansion might have led to population structure relevant for conservation management of the species, for example, in the framework of relocation or reintroduction programs (Mucci et al., 2010). In the first comprehensive genetic assessment across the mainland of Britain, Stanton et al. (2014) describe strikingly different genetic structure in northern compared to southern portions of the island, which they link to different demographic histories for the otters in those regions. More recently Thomas et al. (2022) combined genetic samples across a time span of 21 years (1993–2014) and assessed temporal change in genetic structure over time during the otter's re‐expansion in the United Kingdom. Interestingly, the genetic structure did not weaken over time and genetic diversity did not increase as hypothesized, which the authors attribute to a potential time lag in genetic compared to demographic recovery but potentially, this could also be the result of some unknown barriers to gene flow among sub‐populations.

Genetic variability of the otter on a European scale has been described to be relatively moderate to low, with populations from the Iberian, Italian, and Scandinavian peninsulas being markedly differentiated from central European populations, which collectively appear to be more homogeneous (Mucci et al., 2010; Randi et al., 2003). However, these studies were based on relatively low sample numbers from individual countries and thus may not have been adequate to discern more fine‐scaled patterns. Austria's geographic location encompasses the eastern end of the Alps and thus may contain potential otter habitat on both the northern and the southern side of this large mountain range. Additionally, Austria represents the western border of historically more stable otter populations from Eastern Europe, such as in neighboring Hungary (Lehoczky et al., 2015), or the Slovak and Czech Republics (Hajkova et al., 2011). Thus far, no extensive genetic data has been available from Austrian otter populations that would allow the evaluation of population structure and genetic variability on a larger scale.

Otters from neighboring Hungary show relatively high genetic diversity (Lehoczky et al., 2015) compared to other central European countries that held continuous otter populations such as France (Geboes et al., 2016), Italy (Buglione et al., 2021), or Germany (Effenberger & Suchentrunk, 1999; Honnen et al., 2011), revealing population structure along main river catchments (Tisza and Danube). We therefore hypothesize that the Austrian otter population contains moderate genetic variability and that geographic features like the Alpine divide or major river catchments are the main drivers in shaping the otter's population structure rather than administrative or biogeographically defined borders.

### 1.3 Spatial distribution of otter individuals along river habitats

The otter's elusive behavior allows for little direct observation and therefore many important ecological parameters essential for effective management and conservation efforts are still poorly understood (Hájková et al., 2009). Female home range lengths along rivers have been reported to range between 7.5 and 20 km, and males between 10 and 34.8 km (Erlinge, 1967; Georgiev, 2007; Kruuk et al., 1993; Néill et al., 2009; Quaglietta et al., 2013; Sittenthaler et al., 2016). Thereby, males often overlap with two to three females while otters of the same sex tend to avoid each other (Kranz, 2000; Kruuk, 2006), but this system can be quite flexible. The drivers of this plasticity are still poorly described but suggested factors favoring more overlap include the degree of relatedness, inter‐ and intraspecific competition, and the availability and spatial distribution of resources (Sittenthaler et al., 2016).

### 1.4 River‐stretch‐based density estimates

As a protected animal, non‐invasive methods are almost obligatory for otter monitoring, and indirect genetic‐based approaches, such as via spraint (scat or anal jelly) sampling, have quickly become preferred for data collection (Sittenthaler et al., 2020 and references therein). For large areas, such as nation‐wide surveys, it is clear that some type of extrapolation must be applied, as it is wholly unreasonable to directly quantify the population size of such an animal over thousands of square kilometers. A relatively simple approach has been to extrapolate based on estimated home‐range sizes, and where feasible, scaled to some predictive environmental variable associated to prey variability. This scaling is necessary as home‐range sizes will vary widely dependent on habitat quality—this was the approach taken by Marnell et al. (2011) where female otter home range sizes (varying in the United Kingdom from just over 2 to over 18 km) were extrapolated across occupied territory in Ireland, scaled to ortho‐phosphate levels as a loose proxy for habitat productivity and thus prey abundance. In Austria, direct abundance estimates in small areas have been used in combination with occupied range estimation via bridge‐checks—with and without scaling to spraint densities (e.g., Holzinger et al., 2020; Kofler et al., 2018; Schenekar & Weiss, 2021). One critical aspect of this work, however, has been the size or length of river habitat surveyed, considering the territorial behavior of the fish otter and the universal problem of edge effects when estimating animal population abundance and resource availability (Manly et al., 2002; Potts et al., 2016). While the modeling of home ranges can be helpful, the dendritic nature of river otter habitat adds a complicating factor that most home‐range calculations have not considered (Murphy et al., 2021). Studies in Austria have varied from using river stretches of as little as 10 to up to 90 km in length (Holzinger et al., 2018; Kofler et al., 2018; Schenekar & Weiss, 2021). While the former is considered by some to be insufficient as it represents the lower end of potential home range sizes, the latter requires a great deal of investment in time to accomplish, exceeding home range sizes by up to five times. An objective evaluation of the efficacy of different surveyed river lengths would potentially provide field researchers with a justification for particular study designs.

### 1.5 Study goals

In this study, we apply molecular genetic data at different scales to support estimation and evaluation of several aspects of the conservation assessment of fish otters in Austria. This includes describing the spatial organization of male and female otters along a number river stretches, estimating local densities to be used in large‐scale extrapolation, evaluating different river survey lengths to guide future monitoring designs, and most importantly, describing genetic structure and diversity on a large scale to evaluate the extent to which this aligns with administrative or biogeographic borders, the currently relevant management units within this EU member state. The results should be universally applicable, especially across all of Europe with respect to demands of the European Habitats Directive.

## 2 MATERIALS AND METHODS

### 2.1 Sampling

Genetic samples were collected in the framework of five independent surveys, contracted by the individual governments of the respective Austrian provinces, namely Carinthia, Styria, Salzburg, and Upper Austria. In Carinthia, two independent surveys, one in 2017 and the other in 2020, were conducted. Sampling design varied among surveys due to being largely dictated by the respective provincial government (Table 1, Figure 1). Collection of samples for the Carinthia 2017 survey were conducted by volunteers, for the other surveys by contracted field workers. The type of non‐invasive samples collected were identical among all projects: Otter spraints that were estimated to be fresher than 24 h. All collectors received training for otter spraint identification and age estimation before collection. If only very few samples along reference stretches were found, spraints estimated to be older than 24 h but fresher than 1 week were also collected. For each collected sample, collection date, GPS location and estimated freshness were recorded. Samples were collected without direct contact of the collector and transferred to a collection tube, either without a conservative agent (survey Carinthia 2017) or with a ammonium‐sulfate‐based conservative buffer (buffer from Sittenthaler et al., 2015). Samples from Carinthia 2017 were transferred to −20°C as soon as possible and stored at −20°C until arrival at the genetics laboratory where they were stored at −80°C until DNA extraction. Samples of the other surveys were transferred onto dry ice immediately after sampling where they were stored until arrival at the laboratory. Then they were stored at −20°C until DNA extraction. Additionally, tissue samples were collected opportunistically of reported dead animals during the Carinthia 2017 survey.

**TABLE 1:** Sampling details on the five surveys from which genetic data were synthesized for this study. Given are the Austrian province in which the survey took place, the survey year, the design of the genetic sample collection, the approximate lengths of the reference stretches (if utilized by the respective survey), the number of sample collection events and the respective time period of the collection event. NA: not applicable.

Province	Survey year	Sampling design	# reference stretches	Lengths of reference stretches	# collection events	Collection period
Carinthia	2017	Point‐wise at monitoring bridges	NA^a^	NA	3	21.2.2017–31.3.2017
						25.4.2017–31.5.2017
						16.10.2017–30.10.2017
Carinthia	2020	Along reference stretches	3	20 km	1	4.2.2020–29.2.2020
Styria	2017/18	Along reference stretches	17 + 2^b^	10 km	1	26.11.2017–25.1.2018
Salzburg	2021	Along reference stretches	7	30 km	1	12.2.2021–30.3.2021
Upper Austria	2021	Along reference stretches	8	30 km	1	9.3.2021–28.3.2021

^a^ Sampling was carried out at 378 predefined monitoring bridges with additional opportunistically collected samples at 453 sites.

^b^ 17 reference stretches along rivers + two pond areas (areas of pond areas: 87.9 and 1074 ha).

### 2.2 DNA extraction, genotyping, and sex identification

DNA extraction from spraint samples and PCR set‐up were carried out in a low‐template DNA clean‐room, separated from tissue DNA extract handling and post‐PCR sample processing. All DNA extractions were carried out as soon as possible after each collection event, for spraint samples using either QIAamp Fast DNA Stool Mini Kit (Qiagen, Hilden Germany) or E.Z.N.A. Stool DNA Kit (Omega Bio‐Tek, Norcross, USA) with minor modification to the manufacturers’ protocols. For QIAamp Kit extractions, final incubation time was extended to 5 mins and final elution volume was 100 μL. For E.Z.N.A. Kit extractions, during Steps 1–5 of the Human DNA Detection protocol, all volumes were downscaled by the factor 1.6 and then continued as recommended with a final elution volume of 100 μL. For both protocols, 100–200 mg starting material was used if the sample was stored without storage buffer and 300–400 mg from samples preserved in storage buffer. Tissue samples were extracted using the DNeasy Blood & Tissue Kit (Qiagen, Hilden, Germany) according to the manufacturer's protocol. All extracts were store at −20°C until further processing. Each extraction batch consisted of a maximum of 11 samples and with each spraint extraction batch, a negative extraction control was processed. For genotyping, 11 microsatellite loci, originally described by Dallas and Piertney (1998) were used, following the protocol of Sittenthaler et al. (2015) with a slightly modified reaction set‐up: Each reaction contained 2.5 μL Qiagen Type‐it Multiplex PCR Master Mix (Qiagen, Hilden, Germany), 0.04 μg/μL bovine serum albumin (Fermentas, St. Leon‐Rot, Germany), 0.17 pmol/μL of each primer and ddH_2_O filled up to a reaction volume of 6 μL. Forward primers were fluorescently labeled and PCR products were loaded onto one of our two in‐house genetic analyzers ABI3130\u00D7L or ABI3500\u00D7L (Thermo Fisher Scientific) for fragment analysis. Selected reference samples were run on both sequencers and positive controls with known genotypes were run with every sequence run to ensure data consistency. Electropherograms were analyzed using GeneMapper 3.7 or 6 (Applied Biosystems). The multiple tube approach of Navidi et al. (1992) was applied to avoid mistyping and to create consensus genotypes of individual PCR replicates. Hereby, a heterozygous genotype was accepted after two independent PCR reactions showing both alleles and a homozygous genotype was accepted after three independent PCR reactions showing the same single allele. An iterative approach was applied during PCR replication to continuously exclude low‐quality samples while maximizing genotyping success for medium‐ to high quality samples, based on the principle of Lampa et al. (2013) and containing six steps. Each step consisted of a triplicate PCR of either, Set1, Set2, or both multiplexes of Sittenthaler et al. (2015) and each sample had to pass a certain amplification success threshold in order to be moved to the next step (Supporting Information 1). If the sample did not pass the quality threshold of the respective step, it was discarded from analysis. Using this approach, each locus was typed at least three times, with a maximum of 15 times and an overall maximum of 36 PCR reactions per sample. With each PCR set‐up at least one positive PCR control (diluted otter tissue extract) and a negative control (ddH_2_O) was processed. A sample was classified as “successfully genotyped” if it had confirmed genotypes at 9–11 microsatellite loci. For successfully genotyped samples, two sex‐markers, namely Lut‐SRY (Dallas et al., 2000) and DBY7Ggu (Hedmark et al., 2004) together with a microsatellite locus (Lut914; Dallas et al., 2000) as a positive control were amplified in a multiplex in triplicates. If sexing was not unambiguously possible after these three PCRs, a second set of triplicates was carried out.

### 2.3 Individual identification, genetic diversity, and genetic structure

Matching genotypes among samples were identified with the software *Cervus* 3.0.7 (Kalinowski et al., 2007) and a collapsed dataset with one representative sample per individual was produced. If multiple samples were assigned to one individual, a random sample containing the most complete genotype was kept. Frequency of allelic dropout (ADO) and false alleles (FA) were calculated using the formula of Broquet and Petit (2004). Due to the iterative genotyping process, more replicates were carried out for medium to low‐quality samples. To receive an unbiased estimate of ADO and FA across all genotyped samples, only the first triplicate PCRs of Set1 and Set2 of each genotyped sample (*N* = 3300 PCRs for each locus) were used for this calculation. The presence of potential Null‐Alleles was reviewed with the software *Micro‐Checker* (Van Oosterhout et al., 2004). Number of alleles, expected (*H*_e_) an observed (*H*_o_) heterozygosity per locus as well as probability of identity (*P*_ID_) and probability of identity for siblings (*P*_IDsibs_) were calculated with *Cervus* and overall unbiased probability of identity (*P*_IDunbias_) were calculated using *Gimlet* v.1.3.3 (Valière, 2002). Factorial Correspondence Analysis was carried out via *GENETIX* 4.05.2 (Belkhir et al., 2004) and visualized in *R* 4.1.2 (RStudio Team, 2020) and *ggplot2* (Wickham, 2016). Genetic cluster identification of genotyped individuals was carried out with in two different programs: First, clusters were identified de novo using the “find. cluster” function of R package *adegenet* 2.1.5 (Jombart, 2008) by using successive K‐means calculation after transforming the data via principal component analysis detection criterion. For this, a maximum of 10 clusters were defined. The best model was chosen based on the “diffNgroup” criterion for automatic selection of clusters. Optimal number of clusters were identified by the Bayesian information criterion (BIC, plot of BIC values of individual cluster numbers: Supporting Information 3). Second, by applying the spatial mixture model of the software *Geneland* (Guillot et al., 2005) for codominant data, accounting for the presence of null alleles and assuming uncorrelated allele frequencies. A total of 1,000,000 iterations were run with a thinning interval of 100. Five independent runs were conducted, each with the number of clusters varying from 1 to 10. Additionally, to infer potential admixed ancestry of individuals, an admixture model was run in *Geneland*, with a total of 100,000 iterations and a thinning interval of 100. For identified cluster via *adegenet*, a Discriminant Analysis of Principal Components (DAPC) was carried out in adegenet, retaining 30 principal components and two discriminant functions. Selection of the number of principal components to be retained was done interactively, using a threshold of 90% explained cumulative variance. Diversity indices (expected and observed heterozygosity, F_IS_‐value, and pairwise F_ST_‐ values among clusters were calculated using *GENEPOP* 1.2 Raymond & Rousset, 1995).

### 2.4 Spatial otter distribution along 30‐km study stretches

To estimate potential differences in male and female marking and ranging behavior, a more detailed analysis was conducted using the data from the 15 reference stretches of 30 km lengths (data from Salzburg and Upper Austria surveys, Supporting Information 2). For individuals identified from these stretches, the number of assigned spraints were calculated. For those individuals having more than one spraint assigned, the “recapture distance” (distance along the main watercourse between the two most distant samples assigned to the same individual) was calculated and the “recapture stretch” (section along the main watercourse in which samples of this individuals were found) was mapped. Of these recapture stretches, the fraction of overlap with the recapture stretches of other individuals of a) the same sex and b) the opposite sex was calculated. This was done separately for males and females as well as overall. To test for statistical differences between males and females of the above‐mentioned parameters (spraint count, recapture distance, and percentages of overlap), Wilcoxon–Rank sum tests were carried out. All statistical analyses were carried out in R 4.1.2 (RStudio Team, 2020) and the package *ggplot2* (Wickham, 2016) was used for plotting. GIS analyses and map construction were carried out in QGIS Desktop 3.22.6 (QGIS Development Team, 2019).

### 2.5 Modeling of density estimations along study stretches

Densities along each of the 15 30‐km‐long study stretches were simulated across sample stretch lengths between 5 and 30 km at 1‐km intervals using a custom resampling algorithm. For each stretch length (repeated for each of the 15 study stretches) 100 random sample stretches were drawn along the full 30 km, and densities on the drawn sample stretches were calculated. Relative density errors (the percentage of over‐ or underestimation compared to the density estimate using the full 30 km stretch) were calculated across all 15 reference stretches. To evaluate whether overall otter density (using the full 30 km stretch) itself was a factor influencing estimation errors, the relative density errors were also regressed on this overall otter density, across the dataset and their correlation evaluated with the non‐parametric Kendall's tau statistic.

## 3 RESULTS

### 3.1 Sampling, genotyping, otter identification, and genetic diversity

Across all five surveys, 2877 spraint samples and 14 tissue samples were collected. Of these, 2492 spraint and all 14 tissue samples were processed in the laboratory. Of the 2492 spraint samples, a total of 1100 was successfully genotyped (success rate 44.1%, Table 2). All 14 tissue samples were successfully genotyped. From the successfully genotyped samples, a total of 384 otter individuals (199 females, 178 males, and 7 of unknown sex) were identified. Five individuals of the Carinthia 2017 survey were again identified from spraint samples in the Carinthia 2020 survey, otherwise no individual was identified from two different surveys. In surveys for which sampling was carried out along reference stretches, no individual was identified from more than one stretch. Of the 73 536 pairwise comparisons among the 384 individuals, all but six pairings had three or more mismatching loci in their genotypes, with these six pairings having two locus mismatches each. Five of these six pairings additionally differed at least at one of these two loci by distinct alleles (and not simply by homozygous/heterozygous variations of the same alleles), and/or differed by sex and/or were more than 50 river‐km separated from each other.

**TABLE 2:** Summary statistics of collected samples and identified otter individuals across the five otter surveys. Given are number of samples collected, number of samples analyzed, number of samples successfully genotyped, and number of otters identified (f: female, m: male, u: unknown sex). Success rate represents fraction of successfully genotyped samples of analyzed samples. The 14 extra samples of the Carinthia 2017 survey represent tissue samples while all other samples are spraint samples.

Project	# collected	# analyzed	# successfully genotyped (success rate)	# fish otters identified (f/m/u)
Carinthia 2017	884 + 14	884 + 14	251 (28.4%) + 14 (100%)	143 (62/76/5)
Carinthia 2020	257	194	103 (53.1%)	27 (13/14/0)
Styria	633	434	239 (55.1%)	80 (42/36/2)
Salzburg	473	473	262 (55.4%)	51 (31/20/0)
Upper Austria	630	507	245 (48.3%)	88 (55/33/0)
Total	2877 + 14	2492 + 14	1100 (44.1%) + 14 (100%)	384 (199/178/7)

The rate of ADO ranged from 13.6% to 32.5% among loci, with a total average of 26.3%. FA were detected on average in 2.3% of PCR replicates (range: 1.1%–3.7%) (Table 3). Overall unbiased probability of identity (*P*_IDunbias_) was 1.51 × 10^−9^. Micro‐Checker Analysis suggested presence of potential Null‐Alleles at seven out of the 11 Loci, which might obscure HWE calculations (Table S2). Factorial Correspondence Analysis showed just very weak to no genetic structure among individual surveys or river catchments (Figure S3).

**TABLE 3:** Summary statistics of the 11 genotyped microsatellite loci. Given are rate of allelic dropout (ADO) and false alleles (FA) for the 1100 scat samples. For genotypes of the 284 identified individuals, the number of alleles (*N*_A_), observed heterozygosity (*H*_O_), expected heterozygosity (*H*_E_), significance of test for deviation of Hardy–Weinberg Equilibrium (***p < 0.001, **p < 0.01, *p < 0.05, NS: not significant; after Bonferroni Correction), probability of identity (*P*_ID_), and probability of identity for siblings (*P*_IDsibs_).

Locus	ADO (%)	FA (%)	*N*_A_	*H*_O_	*H*_E_	HWE	*P*_ID_	*P*_IDsibs_
Lut435	13.6	1.6	8	0.380	0.501	***	0.281	0.570
Lut457	31.3	2.7	6	0.645	0.682	NS	0.164	0.451
Lut615	32.2	3.7	8	0.630	0.698	**	0.143	0.437
Lut701	31.1	2.7	6	0.676	0.775	*	0.083	0.384
Lut717	32.5	2.1	7	0.638	0.751	*	0.105	0.401
Lut833	26.3	1.5	9	0.480	0.647	***	0.186	0.473
Lut453	20.8	4.1	6	0.597	0.686	**	0.157	0.447
Lut604	21.7	1.9	6	0.544	0.653	***	0.183	0.470
Lut715	30.5	1.5	5	0.624	0.640	NS	0.198	0.480
Lut733	25.6	2.4	5	0.665	0.662	NS	0.169	0.462
Lut832	27.8	1.1	6	0.649	0.670	***	0.164	0.456
overall	26.3	2.3	6.55	0.593	0.669		1.69 × 10^−9^	1.74 × 10^−4^

**FIGURE 2:**
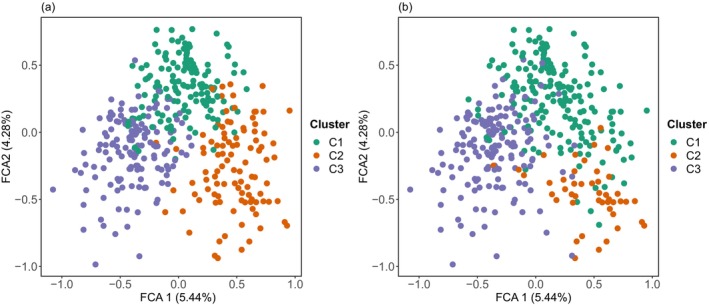
Factorial Correspondence Analysis of the 384 identified otter individuals, color coded by the identified clusters of adegenet (a) and Geneland (b).

**FIGURE 3:**
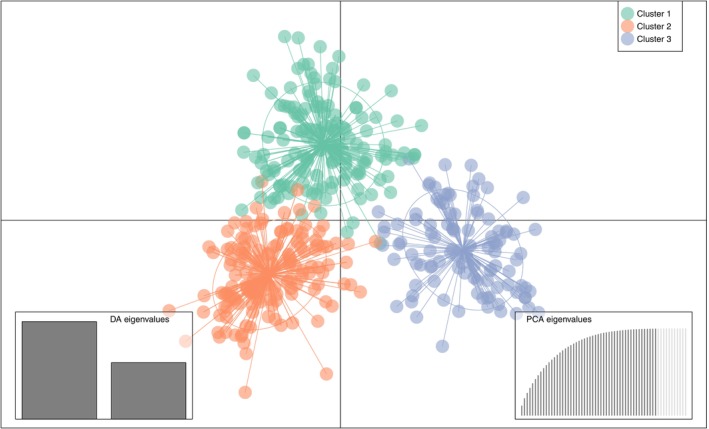
Scatterplot of Discriminant Analysis of Principal Components (DAPC) of the 384 identified otter individuals assigned to the three identified clusters by *adegenet*. Inertia ellipses and star lines indicate center of respective clusters.

Both cluster identification analyses suggested a most likely number of clusters of three. Assigned clusters were largely congruent between the two methods with few individuals having different assignments (with both cluster‐sets showing relatively clear but overlapping clusters of individuals based on the FCA; Figure 2). DAPC assigned 97.4% of the individuals correctly to the identified clusters (99.3%, 97.1%, and 95.8% for cluster 1–3, respectively) and showed relatively clear separation of the three suggested clusters (Figure 3).

**FIGURE 4:**
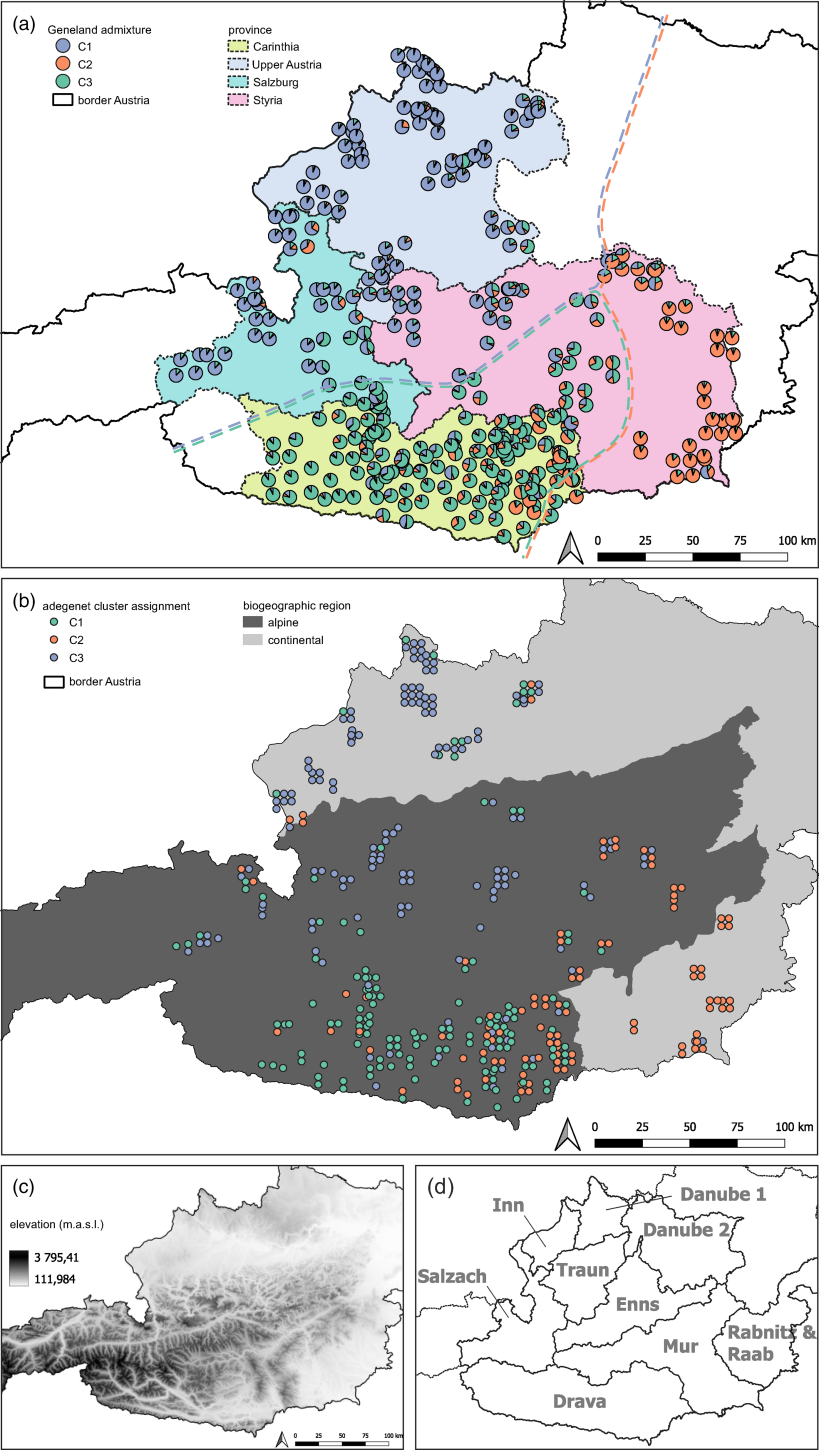
Results of admixture analysis from *Geneland* and cluster identification analysis from *adegenet*. Each datapoint indicates one representative sample for each of the 384 otter individuals identified. (a) Pie charts showing the proportional assignments of each individual to the three identified clusters from *Geneland* admixture analysis in relation to provincial borders. Colored dashed lines indicate borders of the geographic expansion of the three genetic clusters suggested by *Geneland*. (b) Most likely cluster assignment from mixture analyses of *adegenet* for each individual in relation to the two biogeographic regions in Austria. In both (a, b) individual data points are slightly scattered for better visualization. (c) Digital elevation model of Austria (m.a.s.l. = meters above sea level) (d) Main river catchments of Austria.

*Geneland* cluster identification showed a clear separation into three geographic areas (Figure 4a, Figure S4): Cluster 1 was only south of the Alpine divide, namely in the Drava and Mur river catchments. Cluster 2 was also confined to the southern side of the Alpine divide (except for 2 individuals, close to the eastern run‐out of the Alps) namely to the Mur and Raaba/Rabnitz catchments and very eastern tip of Enns and Drava catchments. Cluster 3 was only found north of the Alpine divide, namely in the Danube, Traun, Inn, Salzach, and Enns River catchments. Cluster assignment from *adegenet* analysis followed this general trend, but with a weaker geographic structure of the cluster assignments (Figure 4b). Particularly Carinthia appeared to be more admixed between cluster 1 and cluster 2 by *adegenet* than by *Geneland* (Figure 4b, Figure S4). All three clusters from both clustering methods showed heterozygote deficiencies (Table 4). Furthermore, all three clusters showed clear genetic differentiation from each other, also using both clustering methods (Table 5).

**TABLE 4:** Genetic diversity of the three identified clusters (C1–C3) from *adegenet* and *Geneland*, respectively. *F*_IS_: *F*_IS_ values *P*_Hedef_: *p*‐value of test for HWE‐deviation by heterozygote deficiency.

	Adegenet			Geneland		
	C1	C2	C3	C1	C2	C3
*H*_O_	0.565	0.623	0.600			
*H*_E_	0.597	0.644	0.633			
*F*_IS_	0.054	0.033	0.052	0.073	0.059	0.077
*P*_Hedef_	<0.001	0.052	<0.001	<0.001	0.014	<0.001

**TABLE 5:** Pairwise differentiation among the three identified clusters (C1–C3) from *adegenet* and *Geneland*, respectively. Values in matrix represent pairwise *F*_ST_ values and *** indicates a *p*‐value <0.001 of exact *G* test for genotypic differentiation. Bottom left half of matrix refer to *adegenet* clusters, upper right half to *Geneland* clusters.

	C1	C2	C3
C1	–	0.103***	0.056***
C2	0.113***	–	0.092***
C3	0.079***	0.119***	–

### 3.2 Individual marking/sprainting behavior

A total of 139 otter individuals were identified along the 15 30‐km‐long stretches in Upper Austria and Salzburg. Otter densities varied from 0.1 to 0.47 otter per river‐kilometer (mean: 0.306, stdev: 0.104; Table 6). Of the 139 otters identified, 42 individuals were only detected from one sample while the remaining 97 were detected from multiple samples with a maximum of 19 samples per individual, whereby the number of assigned samples per individual showed a Poisson distribution (Figure S5). Males showed a higher overall number of assigned samples (mean: 4.85, median: 3) than females (mean: 2.9, median: 2), though this was not statistically significant (*W* = 1863, *p* = 0.066).

**TABLE 6:** Details on the identified otters at the 15 30‐km reference stretches. Given are the respective code for each stretch, the stretch name, the respective length in km, the number of total otters identified, as well as number of males and females. Furthermore, the resulting sex ratio (males to females) as well as the otter density (in otters per river‐kilometer) are given.

Stretch code	Stretch name	Length (km)	# otters identified	# males	# females	Sex ratio m:f	Otter density (otters/ river‐km)
A	Salzach 1	30.33	8	2	6	0.3	0.264
B	Saalach	30.90	10	4	6	0.7	0.324
C	Großarlbach	29.72	6	3	3	1.0	0.202
D	Mur	29.85	6	3	3	1.0	0.201
E	Taurachbach	29.75	3	2	1	2.0	0.101
F	Lammer	30.23	7	3	4	0.8	0.232
G	Salzach 2	30.68	11	3	8	0.4	0.359
H	Antiesen	29.91	9	3	6	0.5	0.301
I	Donau	31.49	14	4	10	0.4	0.445
J	Enns	29.30	6	3	3	1.0	0.205
K	Große Mühl	29.91	14	4	10	0.4	0.468
L	Traun 1	30.33	13	5	8	0.6	0.429
M	Traun 2	29.64	12	4	8	0.5	0.405
N	Waldaist	30.90	12	6	6	1.0	0.388
O	Schwemmbach	29.55	8	4	4	1.0	0.271

**FIGURE 5:**
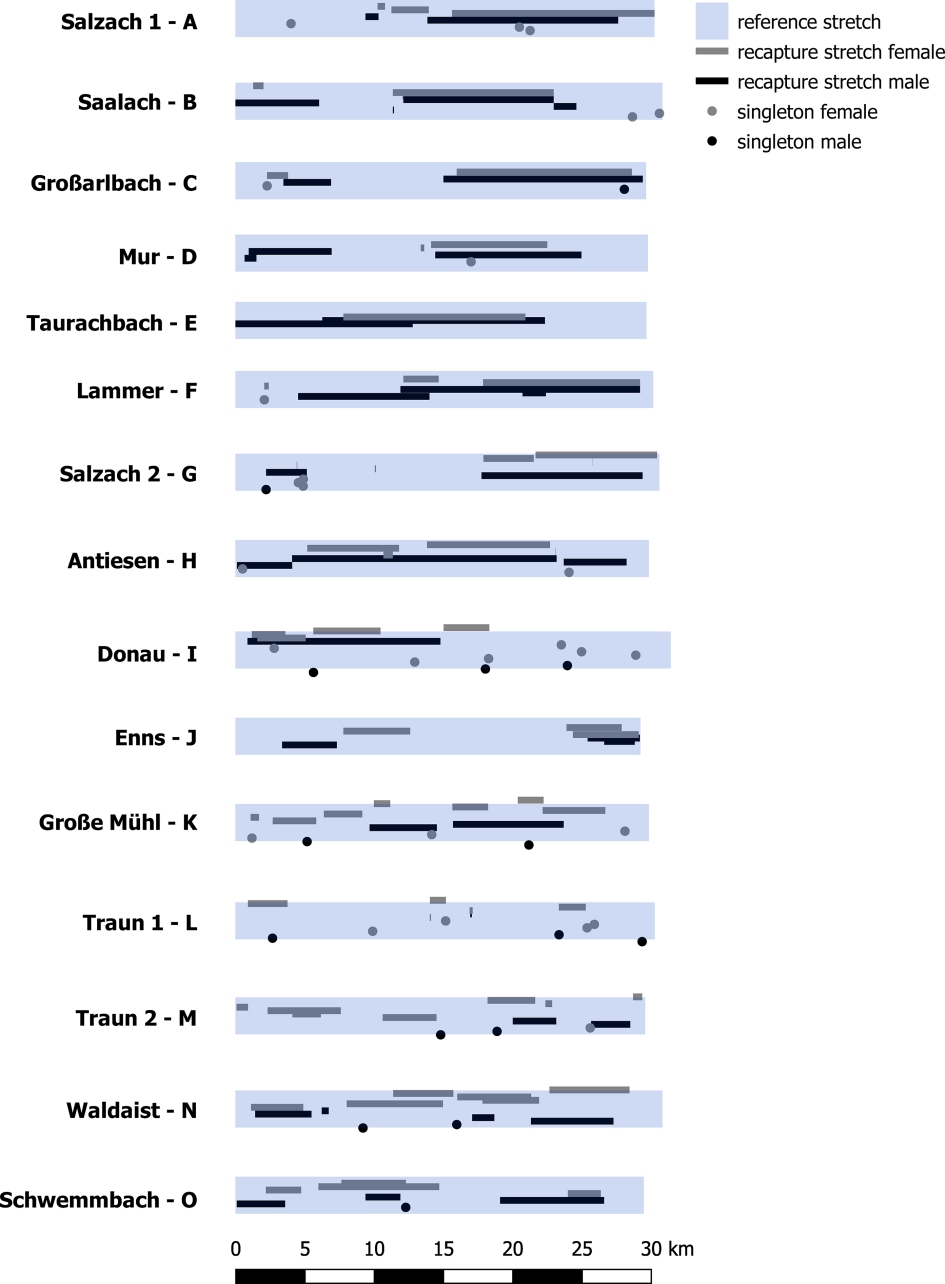
Spatial distribution of the 139 identified otters along the 15 30‐km‐long reference stretches. Sample position of individuals identified by only one sample (“singletons”) are indicated by circles, whereas “recapture stretches” (stretch between the two most distant samples assigned to the same individual) are indicated by rectangles. Details on the reference stretches are given in Supporting Information 2.

Among the 97 individuals identified by at least two samples, the mean recapture distance was 4758 m (stdev: 4506 m, median: 3493 m), with males (mean: 6232 m, stdev: 5305 m, median 4035 m) having significantly longer recapture distances than females (mean: 3809 m, stdev: 3601 m, median 2858 m) (*W* = 823, *p* = 0.028) (Figure S6). Overall, otters showed extensive overlap with recapture stretches of individuals of the other sex, while avoiding individuals of the same sex (Figure 5). In total, the percentage of overlap of recapture stretches with those of other individuals of the same sex was 14.4% while the overlap with individuals of the other sex was 59.2%, a difference that was highly significant (*W* = 6482.5, *p* < 0.001). However, no statistically significant difference could be observed between males and females, neither concerning same‐sex nor other‐sex overlaps (*W* = 1145 and 1113, respectively, both *p* > 0.05, Table 7).

**TABLE 7:** Mean percentage of overlap of recapture stretches for males, and all individuals summed. The two columns indicate overlaps of recapture stretches with recapture stretches of other individuals of the same sex (% overlap same sex) and with recapture stretches of individuals of the opposite sex (% overlap other sex). Italic symbols between numbers indicate statistical significance (sign: *p* < 0.001, n.s.: not significant).

	% overlap same sex		% overlap opposite sex
Males	11.05	sign.	62.26
	n.s.		n.s.
Females	17.87	sign.	55.90
Total	14.37	sign.	59.16

### 3.3 Modeling errors of density estimations along study stretches

**FIGURE 6:**
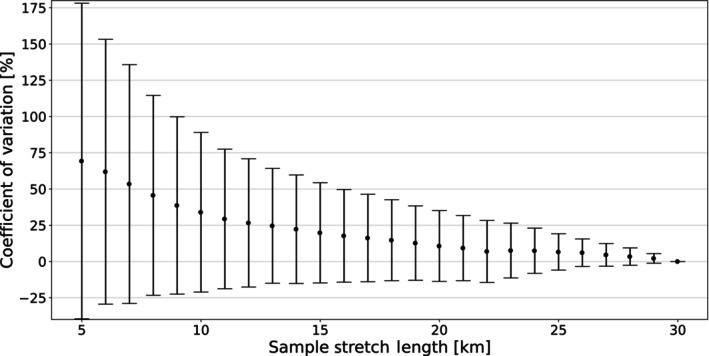
Relative density error (shown as the coefficient of variation) of the calculated otter density for the respective sample stretch length. Error bars indicate ± one standard deviation. One outlier stretch (Taurachbach) is not shown.

**FIGURE 7:**
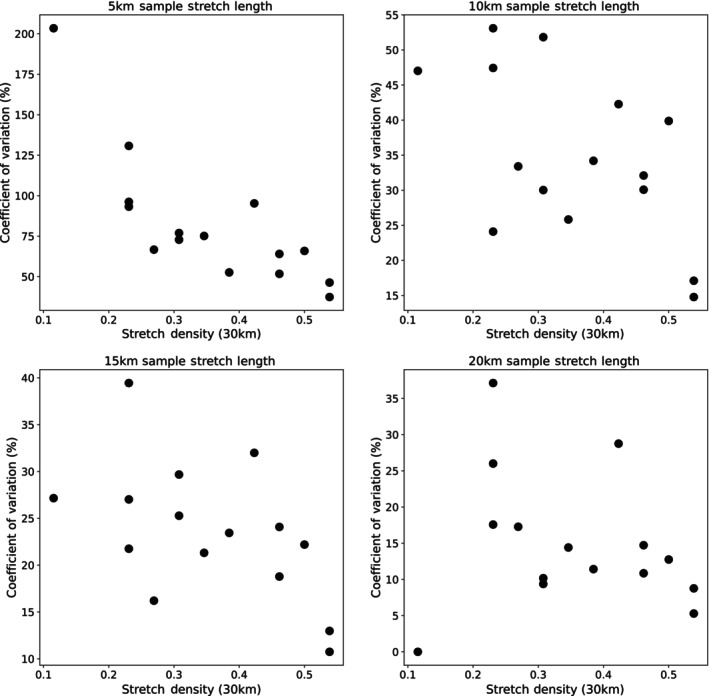
Relationship between relative density error (shown as the coefficient of variation) and the overall density across 30 km. Relationships are shown separately for four different sample stretch lengths (5, 10, 15, and 20 km, respectively).

Across all 15 reference stretches, the relative density error was positive (supporting overestimation) across all sample stretch lengths, though relatively stable at about 20 km or more (Figure 6). The mean relative density error at a sample stretch length of 20 km or greater was less than 10%, supporting that density overestimation, though present, was negligible. The error bars also overlap into the negative range demonstrating that underestimation is also possible across all sample stretch lengths. Additionally, relative errors appeared to be inversely related to density estimates, with the highest errors occurring at the lowest densities (Figure 7). The correlation between otter density and relative errors was negative at all sampled stretch lengths (5, 10, 15, and 20 km), but was not always statistically significant. Removing the one extreme outlier, Taurachbach, with an otter density just over 0.1 km/otter, resulted in three of the four sampled stretch lengths (10, 15, and 20 km) showing a statistically significant Kendall's tau (*p* = 0.017 at 15 km), or borderline significant (*p* = 0.053 and *p* = 0.68 for 10 and 20 km stretches, respectively) value.

## 4 DISCUSSION

In this study, we juxtapose the genetic structure and diversity of a European Habitats Directive listed species to their current management units. We describe genetically determined individual densities as well as the distribution of individuals along Austrian river courses and estimate the risk of error in such density estimates to aid future survey design.

### 4.1 Discrepancy between genetic clusters and management units of a protected species

Our dataset reveals a clear mismatch between the genetically identified clusters and current management units, which are to date solely defined by administrative borders and biogeographic regions. Three clearly distinct genetic clusters were identified within Austria. Their differentiation among each other is slightly lower but comparable to those among the British populations reported by Thomas et al. (2022) (mean *F*_ST_: 0.094 for Austrian clusters, mean global *F*_ST_ among British River Basin District regions: 0.125). The genetic structure revealed by this study does not follow administrative (i.e., provincial) or biogeographic borders and river catchments seem to have only a limited effect on the genetic structure. The lack of such a structure at provincial or biogeographic scales supports our initial hypothesis and highlights a clear difference between genetically identified units and current management units. For example, within Carinthia, one of the two Austrian provinces that ratified a decree for a regulated otter culling, two genetically distinct clusters have been identified. On the other hand, Upper Austria, which falls into both the alpine and the continental region (and therefore needs to apply potential management measures separately for those two biogeographic regions) reveals only one homogeneous population throughout its whole area. Additionally, both Upper Austria and Salzburg share borders with the state of Bavaria in Germany, which is also currently considering culling measures for the otter, based on their own ongoing population estimates. The obvious question to pose, biologically, is whether the fish otter population in Bavaria is shared or distinct with the fish otter population in Salzburg or Upper Austria.

The relatively weak genetic structure revealed along main river catchments in Austria supports relatively high mobility of otters across watersheds (at least across generations), particularly at lower altitudes encompassing less steep terrain. The main geographical feature that shaped genetic structure in Austria was the main Alpine divide, representing a clear colonization barrier. A similar pattern was also observed in Hungary by Lehoczky et al. (2015) where significant structure between the two main river catchments (Tisza and Danube) was detected but not among the smaller sub‐catchments of the area. In France, only weak genetic divergence could be observed between populations from the Atlantic coast and from the Massif Central (Geboes et al., 2016). In southern Italian populations, significant differentiation was observed only partly among river basins (Buglione et al., 2021). Stanton et al. (2014) observed notable population structure in otter populations of southern Britain, whereas there was little to no population structure in northern Britain. This was attributed largely to differing human population density and land use patterns.

Two of the three clusters identified among Austrian otters could reflect the two refuge areas during the 1990s (Cluster 3: refuge area A and Cluster 2: refuge area B, Figures 1 and 4) resulting from presumably anthropogenically caused fragmentation in the mid‐20th century. However, the origin of Cluster 1 is less clear. It might stem from an independent recolonization source, entering Carinthia either along the Drava or from a more southwestern source in Italy. However, since Carinthia is separated from Italy by basically continuous mountain ranges and otter presence has only been reported very recently in the northern parts of Italy (Buglione et al., 2021), the latter is less likely. Therefore, the two genetic clusters south of the Alps might stem either from two different genetic clusters from the Slovenian otter population, or distinct refugia within Austria that escaped notice.

The importance of incorporating genetic information to identify management units for the protection of endangered species has been clearly recognized (Frankham, 2010; Hohenlohe et al., 2021; Segelbacher et al., 2010). Theoretically, the primary benefits of recognizing genetic structure in conservation management most often come into play in fragmented populations, where the risks of inbreeding depression are high. The avoidance of outbreeding depression can also be a concern in relocation or reintroduction efforts. However, practical considerations are also of high importance if culling measures are implemented based on assumed or estimated population growth rates and sizes—when the populations themselves have not been genetically circumscribed. Clearly, species management based on administrative rather than biological borders is not science‐based and thus must be discouraged. Whether a species is heavily managed or not, a better alignment between science and conservation practice across different administrative regions is required to enhance species recovery plans as illustrated by an example of the lilypad whiteface *Leucorrhinia caudalis* in Switzerland that is listed in Annex IV of the Habitats directive (Keller et al., 2010, 2015). Finally, we would like to add that we did observe gene flow among the three identified clusters (as seen by admixed individuals, e.g., in central Styria, Figure 4a), illustrating a reconnection of the presumably once isolated clusters, which is, from a conservation genetic perspective naturally a positive development.

### 4.2 Genotyping, otter identification, and genetic diversity

Overall, described error rates (ADO, FA) and genotyping success rates fall within the range of previous studies (see Supporting Information 5 for further discussion).

The genetic diversity of the Austrian otters (*H*_O_ = 0.59, *H*_E_ = 0.67, *N*_Aavg_ = 6.55) is lower than that of the otter population in Hungary (*H*_O_ = 0.67, *H*_E_ = 0.71, *N*_Aavg_ = 7.25; Lehoczky et al., 2015), lower than those of most of the identified genetic groups in Fennoscandia (Honnen et al., 2011), comparable to the genetic diversity of a population from North‐East Spain (Ferrando et al., 2008) or Germany (Lampa et al., 2015) but higher than those described from population in Italy (*H*_O_ = 0.45, *H*_E_ = 0.47; Buglione et al., 2021). It furthermore fell into the middle of the range reported from European populations by Randi et al. (2003) and from British populations by Thomas et al. (2022) and in the upper half of the range reported by Mucci et al. (2010). While the majority of these studies drew from the same set of microsatellite loci originally described by Dallas and Piertney (1998), the exact number and set of loci utilized varied among studies and thus these comparisons should be interpreted with caution. Nevertheless, in summary, Austrian populations seem to exhibit a medium to high genetic variability compared to other European otter populations. Austrian otters show higher genetic variability compared to small populations that were completely or nearly depleted during the 20th century and a lower variability compared to larger eastern or northern European populations that persisted throughout the general population decline.

Sex ratio (ratio of identified female individuals to male individuals) in our dataset was relatively even overall, however we found a female‐biased sex ratio in the two most recent studies, namely in Salzburg (m:f ratio 1.55; *N* = 51) and Upper Austria (m:f ratio 1.67; *N* = 88). Whether this represents a temporal trend between 2017 and 2021 or is rather a spatial pattern, needs to be evaluated. Two independent sex‐markers as well as a positive control were typed for sex determination, rendering laboratory errors unlikely.

### 4.3 Modeling otter densities at stretches

At a smaller scale, our simulation results provided some reflection on the effects of stretch length for the genetic‐based density estimations along river corridors. While mean relative errors supported a general tendency of overestimation, the degree of overestimation was negligible at the individual stretch level (i.e., mostly less than 1 otter). For landscape‐wide extrapolation, a reduction of approximately 10% would be warranted when sample stretches of 20–30 km in length are used. Nonetheless we note that typical 95% CIs of such landscape‐wide abundance estimates were already at plus or minus 20% of mean estimates (e.g., Holzinger et al., 2018; Schenekar & Weiss, 2021), and thus would not influence the overall estimates in any significant way. Evaluation of the potential increase in accuracy for sample stretch lengths above 30 km in length was not possible in our study, as the relative error automatically converges to zero at the maximum length of the sample stretch (in our case 30 km). To estimate potential edge effects at 30 km, sample stretches of at least 35–40 km would be necessary. Overall, considering the many factors contributing to relatively large confidence intervals for landscape‐wide otter abundance estimates, we consider sample stretch lengths of 25 or 30 km sufficient to satisfy conservation management goals, at least at the range of densities reported in this study.

### 4.4 Spatial otter distribution along river habitats

Genetic data was also valuable in identifying meaningful patterns of distribution within sample stretches. Male otters clearly deposited more spraints than females, reflected by the overall higher number of spraints assigned per individual to males, a phenomenon described in a study from Germany (Lampa et al., 2015) but interestingly not in a study from northeast Austria (Sittenthaler et al., 2020). Furthermore, male otters had overall significantly larger recapture distances, presumably mirroring the males’ larger home range sizes (Erlinge, 1967; Kruuk, 2006; Néill et al., 2009; Quaglietta et al., 2013). We would like to emphasize that our calculated recapture distances are not to be interpreted as absolute otter home ranges as our survey designs relied on single sampling events along linear habitats. Our recapture distances may be better understood as “minimum home ranges” as described in Sittenthaler et al. (2015), but since these authors carried out a more exhaustive sampling strategy, also incorporating distinction between resident and putatively transient individuals, we prefer to keep a separate terminology and refrain from direct comparisons of absolute values.

Finally, the limited overlap of the recapture distances of same‐sex individuals (14.3%, vs. 59.16% overlap of different‐sex individuals) is also reflective of what has been described previously (Kranz, 2000; Kruuk, 2006) but we did see great variability in range overlap at the individual level (Figure 5). The question whether increased overlap of different‐sex individuals stems from higher relatedness of the involved individuals or is promoted by more favorable environmental conditions (e.g., increased food or resting site availability) falls outside the scope of this study. However, incorporating additional environmental data into the dataset and investigating the dependency of otter densities, percentage of home range overlaps or recapture distance lengths from such environmental conditions, opens an interesting avenue for future research.

In summary, this work demonstrates how the application of genetic data can support the evaluation of various biological attributes of a species relevant for conservation assessment and management. In particular, we highlight the incompatibility of administrative or biogeographically circumscribed management units with population genetic structure and point out that this is the status quo for conservation management among member states of the European Union. We advocate more focused integration of genetic structure into population assessment and especially management planning. Within the European context, there need not be any change to legislation to facilitate more science‐based decisions, but rather more communication and cooperation across administrative borders be it within or among EU member states.

## CONFLICT OF INTEREST STATEMENT

The authors declare no conflicts of interest.

## Supporting information


Data S1.

Data S2.

Data S3.

Data S4.

Data S5.

Data S6.

## Data Availability

The raw data underlying the main results are available as supplementary material (Supporting Information 6) to this study. Data Accessibility: Sampling locations and dates, microsatellite and sexing data are all included in Supplementary Information 6. Benefits generated: All authors are either citizens or active researchers in Austria and the data generated resulted from a multilateral collaboration among provincial governments and the University of Graz. The gained insights can be used for future fish otter conservation and management in Austria.
