# Supplementary material for: Applying molecular genetic data at different scales to support conservation assessment of European Habitats Directive listed species: A case study of Eurasian otter in Austria
Source: Evol Appl. 2023 Sep 27;16(10):1735–52. doi: 10.1111/eva.13597 (PMC10660814; doi:10.1111/eva.13597)
Supplement: Supplementary file 1 — Data S1. [file EVA-16-1735-s002.pdf]

Supporting Information 1

from

Applying molecular genetic data at different scales to support conservation assessment of European Habitat Directive listed species: a case study of Eurasian otter in Austria

Journal: Evolutionary Applications

Workplan for otter spraint PCRs: modified from Lampa et al. (2013)

Rules to accept a genotype:
Heterozygous: Same genotype detected in at least 2 independent PCR reactions
Homozygous: Same genotype detected in at least 3 independent PCR reactions

Step 1: PCR of Set2 in triplicate

- Amplicons in less than 30% of PCRs: Discard sample
- Amplicons in more than 30% in PCRs: move sample to Step 2

Step 2: PCR of Set1 in triplicate

- Amplicons in less than 30% of PCRs overall: Discard sample
- Amplicons in more than 30% in PCRs overall: move sample to Step 3
- Genotype complete: sample done

Step 3: First repetition to complete genotypes (Set1 or 2 Set2 or both, in triplicates)

- Less than 4 Loci confirmed: Discard sample
- 7 to 1 loci missing: proceed to step 4
- Genotype complete: sample done

Step 4: Second repetition to complete genotypes (Set1 or 2 Set2 or both, in triplicates)

- Less than 7 loci confirmed: discard sample
- 4 to 1 loci missing: proceed to step 5
- Genotype complete: sample done

Step 5: First round of individual loci PCR to complete genotypes (in triplicates)

- More than 2 loci missing: discard sample
- 1 or 2 loci missing: proceed to step 6

- Genotype complete: sample done

Step 6: Second round of individual loci PCR to complete genotypes (in triplicates)

- Sample done (either 1, 2 or zero loci missing)

References:

Lampa S, Henle K, Klenke R, et al (2013) How to overcome genotyping errors in non-invasive genetic mark-recapture population size estimation - A review of available methods illustrated by a case study. J Wildl Manage 77:1490–1511. https://doi.org/10.1002/jwmg.604
