# Supplementary material for: Applying molecular genetic data at different scales to support conservation assessment of European Habitats Directive listed species: A case study of Eurasian otter in Austria
Source: Evol Appl. 2023 Sep 27;16(10):1735–52. doi: 10.1111/eva.13597 (PMC10660814; doi:10.1111/eva.13597)
Supplement: Supplementary file 2 — Data S2. [file EVA-16-1735-s006.pdf]

Supporting Information 2

from

Applying molecular genetic data at different scales to support conservation assessment of European Habitat Directive listed species: a case study of Eurasian otter in Austria

Journal: Evolutionary Applications

Details on the 15 30 km reference stretches.

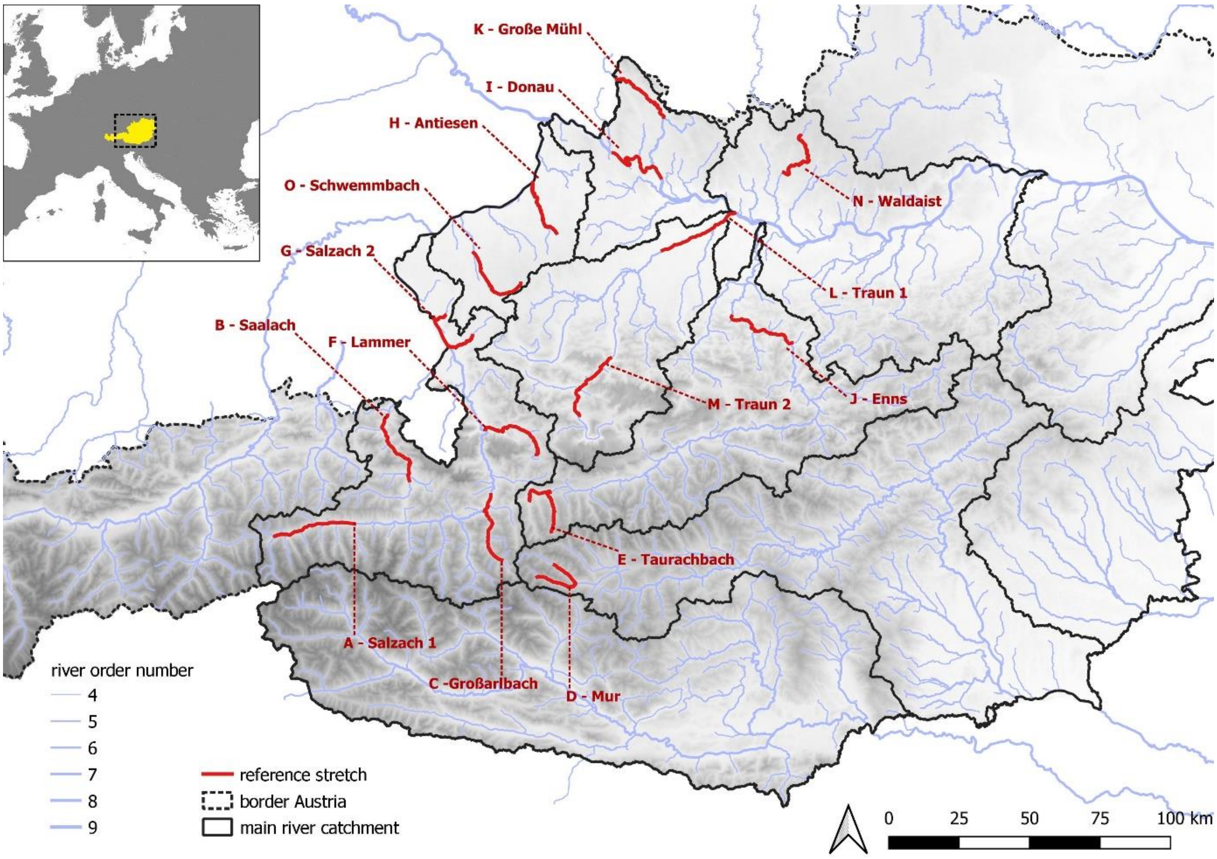

Fig. S1 Relative position of the 15 reference stretches in the Austrian river network. Labels refer to stretch codes and stretch names of Table S1. Thickness of blue line indicates river order number of the respective rivers of the river network in Austria.

Table S1 Geographic details on the 15 reference stretches. Given are the respective stretch code and name used in this manuscript, the total length of the reference stretch. Furthermore, the river order numbe, or if encompassing multiple orders, of the respective section (with the section length) are listed. GPS coordinates of the two endpoints for each stretch refer to WGS84 (EPSG: 3857). Elevation of the respective end points are given in meters above sea level (m.a.s.l.). Finally, biogeographic region is listed.

stretch code	stretch name	length (km)	river order number	GPS coord. end point	GPS coord. end point 2	elevation end point 1 (m.a.s.l)	elevation end point 1 (m.a.s.l)	biogeogr. region
A	Salzach 1	30.33	6 & 5	12.56998, 47.27976	12.19679, 47.23721	772	905	alpine
B	Saalach	30.90	6 & 5	12.72178, 47. 62711	12.82643, 47.41978	549	723	alpine
C	Großarlbach	29.72	7 & 5	13.20987, 47.37480	13.26167, 47.16656	557	1020	alpine
D	Mur	29.85	4 & 3	13.42716, 47.11231	13.50809, 47.15138	1149	1529	alpine
E	Taurachbach	29.75	4 & 3	13.39249, 47.35677	13.49403, 47.25693	882	1298	alpine
F	Lammer	30.23	5	13.18245, 47.58346	13.42477, 47.50399	472	787	alpine
G	Salzach 2	30.68	7 & 4	12.99143, 47.94778	13.12166, 47.88330	407	504	continental
H	Antiesen	29.91	4 & 3	13.40843, 48.37173	13.52199, 48.21074	315	450	continental
I	Donau	31.49	9	14.02309, 48.38548	13.79553, 48.46612	265	281	continental
J	Enns	29.30	6	14.35314, 47.94086	14.63765, 47.85266	331	371	alpine
K	Große Mühl	29.91	5	14.03785, 48.57920	13.81385, 48.70099	495	615	continental
L	Traun 1	30.33	6	14.37450, 48.27010	14.02667, 48.15336	251	310	continental
M	Traun 2	29.64	5	13.77569, 47.81104	13.62823, 47.62878	422	503	alpine
N	Waldaist	30.90	4	14.61161, 48.39842	14.69601, 48.51493	418	770	continental
O	Schwemmbach	29.55	4	13.126075, 48.14747	13.35163, 48.05060	419	588	continental

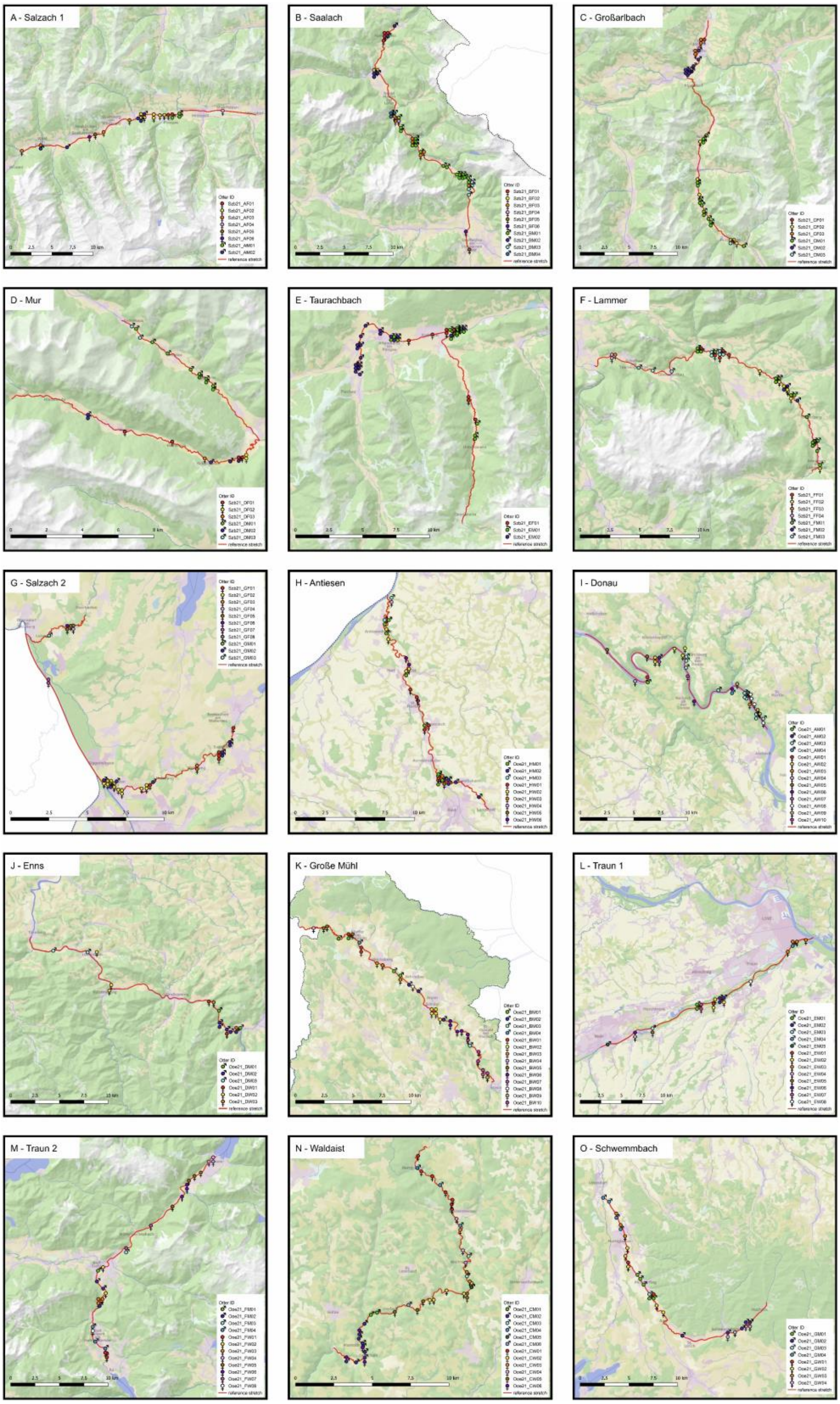

Fig. S2 Detailed view on the 15 30 km reference stretches and location of the successfully genotyped samples. Samples are color coded for the assigned otter individual and shape indicate sex of the individual. Background: Corine Land Cover 2018.
