# Supplementary material for: Applying molecular genetic data at different scales to support conservation assessment of European Habitats Directive listed species: A case study of Eurasian otter in Austria
Source: Evol Appl. 2023 Sep 27;16(10):1735–52. doi: 10.1111/eva.13597 (PMC10660814; doi:10.1111/eva.13597)
Supplement: Supplementary file 3 — Data S3. [file EVA-16-1735-s004.pdf]

Supporting Information 3

from

Applying molecular genetic data at different scales to support conservation assessment of European Habitat Directive listed species: a case study of Eurasian otter in Austria

Journal: Evolutionary Applications

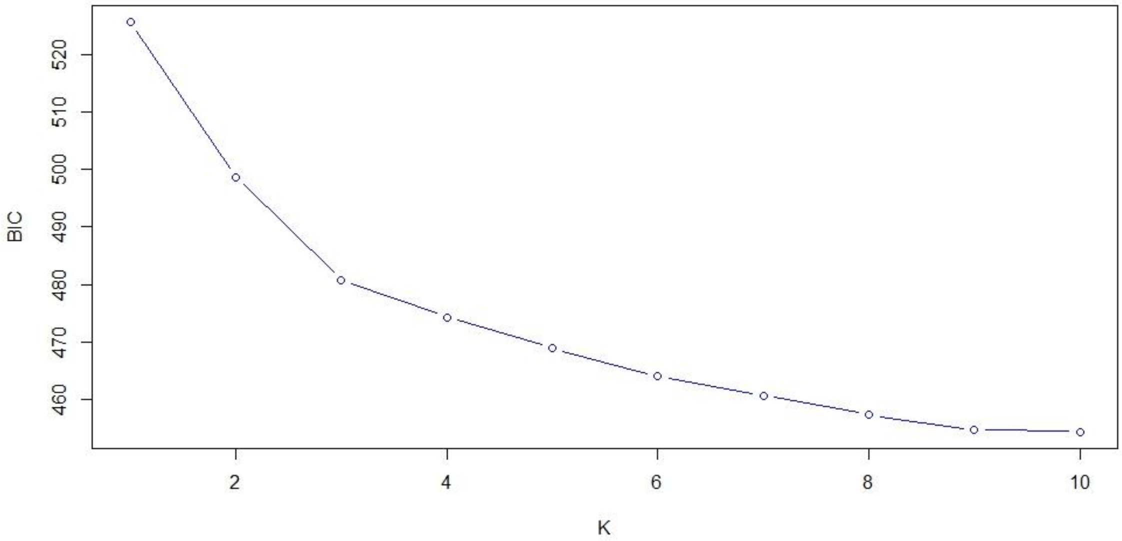

Fig. S3 Bayesian Information Criterion (BIC) scores of the individual number of clusters (K) set from 1 to 10 in the find.clusters() function of adegenet.
