# Supplementary material for: Applying molecular genetic data at different scales to support conservation assessment of European Habitats Directive listed species: A case study of Eurasian otter in Austria
Source: Evol Appl. 2023 Sep 27;16(10):1735–52. doi: 10.1111/eva.13597 (PMC10660814; doi:10.1111/eva.13597)
Supplement: Supplementary file 4 — Data S4. [file EVA-16-1735-s003.pdf]

Supporting Information 4

from

Applying molecular genetic data at different scales to support conservation assessment of European Habitat Directive listed species: a case study of Eurasian otter in Austria

Journal: Evolutionary Applications

Table S2 Output of Micro-Checker analysis, assessing the final dataset for potential Null Alleles.

Locus	Null Present	Oosterhout	Chakraborty	Brookfield 1	Brookfield 2
Lut435	yes	0.1244	0.1357	0.0796	0.0796
Lut457	yes	0.045	0.0512	0.04	0.0708
Lut615	yes	0.0741	0.0806	0.0639	0.2141
Lut701	yes	0.1198	0.1438	0.0979	0.1541
Lut717	yes	0.0785	0.0903	0.0654	0.1079
Lut833	no	0.012	0.0152	0.012	0.0319
Lut453	no	0.0284	0.027	0.0213	0.0213
Lut604	yes	0.0639	0.0678	0.0554	0.0554
Lut715	yes	0.0632	0.0691	0.0525	0.1084
Lut733	no	0.0113	0.0125	0.0096	0.0314
Lut832	no	-0.0053	-0.003	-0.0024	0.0236

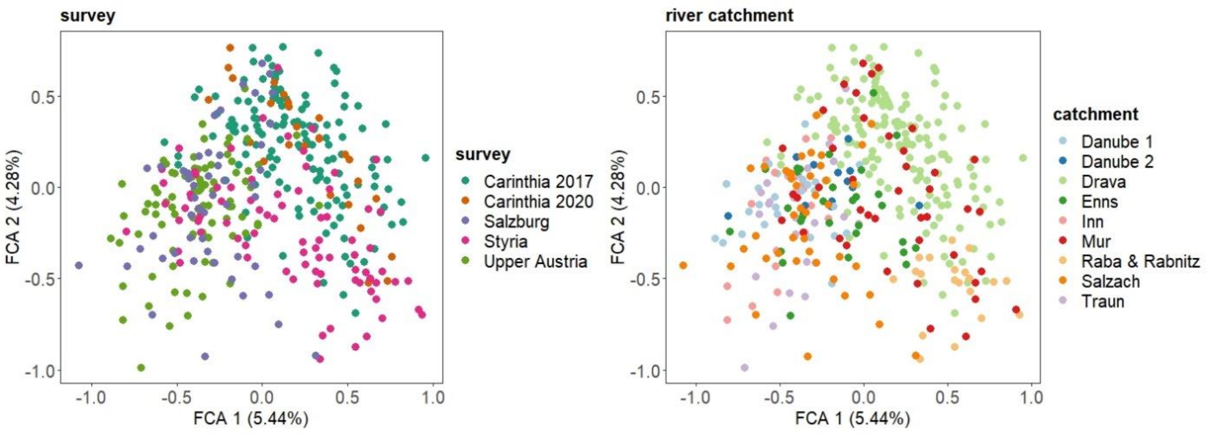

Fig. S3 Factorial Correspondence Analysis of the 384 identified otter individuals, color coded by survey (left) and river catchment (right).

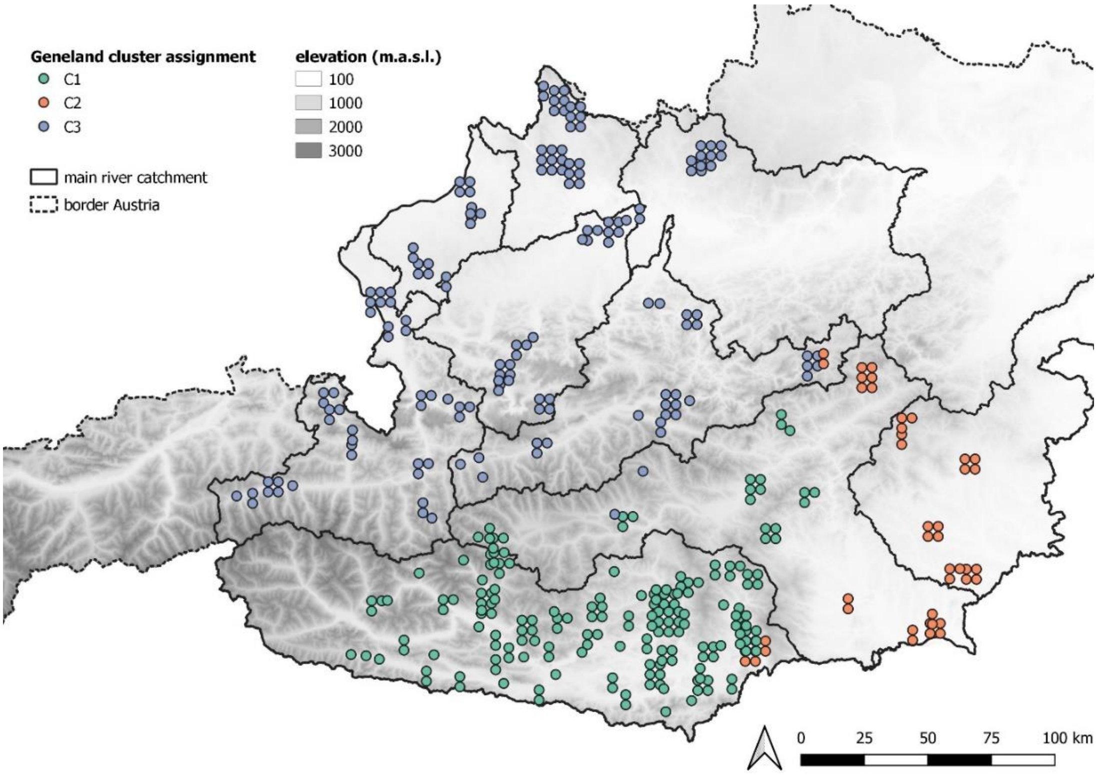

Fig. S4 Results from *Geneland* cluster identification and assignmentsprojected onto the study area. Each datapoint represents a representative sample of each of the 384 identified individuals. Color codes indicate most likely cluster assignment for each individual. Background is color coded for elevation (m.a.s.l. = meters above sea level). Black lines indicate main river catchments of the area. Individual data points are slightly scattered for better visualization.

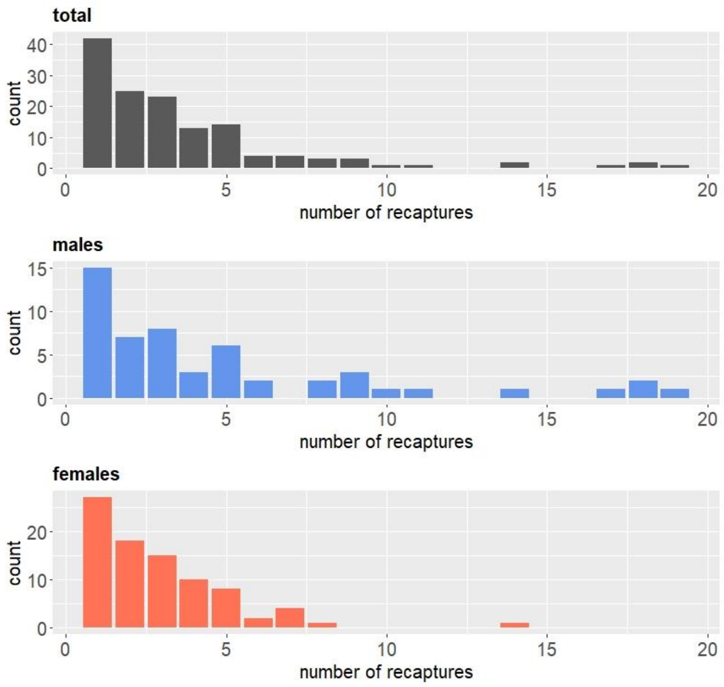

Fig. S5 Frequencies of numbers of assigned samples per individual (=" number of recaptures") of all 139 individuals of the 15 30 km stretches (total), of males only (males) and of females only (females).

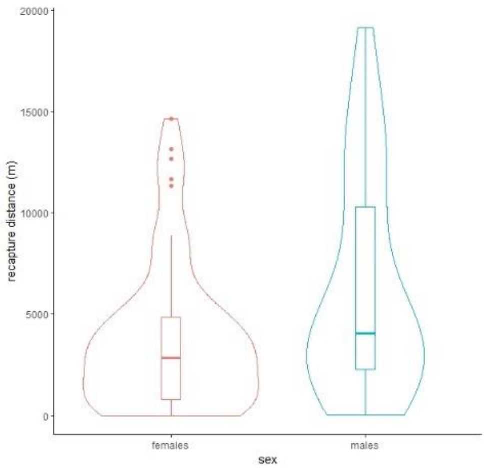

Fig. S6 Boxplot-violin plots of the recapture distances of females and males.
