# Supplementary material for: Applying molecular genetic data at different scales to support conservation assessment of European Habitats Directive listed species: A case study of Eurasian otter in Austria
Source: Evol Appl. 2023 Sep 27;16(10):1735–52. doi: 10.1111/eva.13597 (PMC10660814; doi:10.1111/eva.13597)
Supplement: Supplementary file 5 — Data S5. [file EVA-16-1735-s005.pdf]

Supporting Information 5

from

Applying molecular genetic data at different scales to support conservation assessment of European Habitat Directive listed species: a case study of Eurasian otter in Austria

Journal: Evolutionary Applications

Genotyping, otter identification and genetic diversity

The total rate of allelic dropout in successfully genotyped samples falls within the range of 19 studies across various mammals (Broquet & Petit 2004), reflecting the relatively high degradation rate in otter scat (Arrendal et al., 2007; Lampa et al., 2013; Lanszki et al., 2008). The rate of false alleles (FA) was relatively low compared to that reported by Broquet & Petit (2004), which might stem from the higher fidelity of the Taq- polymerase used in this study. The success rate of genotyped samples (44.1%) falls into the center of the range reported by Hájková et al. (2009) (14-73%) and is comparable to the success rate of Sittenthaler et al. (2021). The success rate of the Carinthia 2017 survey (28.4%) was notably lower than those of the other Austrian surveys (48.3-55.4%), presumably due to the use of volunteers and dry strong in that survey. For subsequent surveys we employed contract biologists, a saline buffer and immediate storage on dry ice. A direct comparison (*N* = 55 samples, data not shown) of the two sample storage options (with conservative buffer and at -20°C versus no buffer and at -80°C) also suggested a slightly higher amplification success with samples stored in the buffer (amplification success 35.7% versus 30.6%).

In non-invasive genetic analyses, insufficient discriminatory power and genotyping errors can lead to under- and over-estimation of the population sizes, respectively (Lampa et al. 2013). The former leads to the so-called shadow effect, resulting in two identical individuals possessing the same genotype. To reduce this risk, a 5- or 6-locus system and an maximum $P_{IDunbias}$ between $1*10^{-3}$ to $1*10^{6}$ has been recommended (Lampa et al., 2013; McKelvey, 2009; Waits et al., 2001) with more conservative values for large populations (>200 individuals) or for high levels of relatedness. Our 11-

locus system revealed an overall $P_{IDunbias}$ of $1.51*10^{-9}$ and thus sufficient discriminatory power. Concerning genotyping errors leading to so-called ghost phenotypes (Lampa et al. 2013), we quantified the rate of allelic dropout (ADO) and false alleles (FA). While ADO was moderately high compared to other studies (see above) we compensated this with a sufficient number of replications in our typing protocol (up to 15 replicates per locus and up to 36 PCR reactions per sample). FA, on the other hand, was comparably low in our dataset. Furthermore, among the 73 536 pairwise comparisons of the 384 identified individuals, all but six differed by three or more loci, making ghost individuals very unlikely.

We found significant signals of Null-alleles in our dataset, which can occur independent of genotyping errors of non-invasive sampling but rather stem from sequence variation in the flanking regions of the microsatellites (Chapuis and Estoup 2007; Rico et al. 2017), potentially affecting quantitative estimates of population differentiation or Hardy-Weinberg-Equilibrium (HWE). In our dataset we observed a clear deviation of HWE across all samples but also within the identified clusters. HWE deviation with a heterozygosity deficit is to be expected in a sub-structured population caused by the Wahlund effect (Wahlund 1928; Garnier-Géré and Chikhi 2013). Therefore, this deficiency could potentially suggest further cryptic sub-structuring within our three observed clusters as also suggested for Hungarian otter clusters (Lehoczky et al. 2015) but should also be interpreted with caution due to the potential presence of Null-alleles in our dataset.

References

Arrendal J, Vilà C, Björklund M (2007) Reliability of noninvasive genetic census of otters compared to field censuses. Conserv Genet 8:1097–1107. https://doi.org/10.1007/s10592-006-9266-y

Broquet T, Petit E (2004) Quantifying genotyping errors in noninvasive population genetics. Mol Ecol 13:3601–3608. https://doi.org/10.1111/j.1365-294X.2004.02352.x

Chapuis M-P, Estoup A (2007) Microsatellite Null Alleles and Estimation of Population Differentiation. Mol Biol Evol 24:621–631. https://doi.org/10.1093/molbev/msl191

Garnier-Géré P, Chikhi L (2013) Population Subdivision, Hardy–Weinberg Equilibrium and the Wahlund Effect. eLS

Hájková P, Zemanová B, Roche K, Hájek B (2009) An evaluation of field and noninvasive genetic methods for estimating Eurasian otter population size. Conserv Genet 10:1667–1681. https://doi.org/10.1007/s10592-008-9745-4

Lampa S, Henle K, Klenke R, et al (2013) How to overcome genotyping errors in non-invasive genetic mark-recapture population size estimation - A review of available methods illustrated by a case study. J Wildl Manage 77:1490–1511. https://doi.org/10.1002/jwmg.604

Lanszki J, Hidas A, Szentes K, et al (2008) Relative spraint density and genetic structure of otter (Lutra lutra) along the Drava River in Hungary. Mamm Biol 73:40–47. https://doi.org/10.1016/j.mambio.2007.08.005

Lehoczky I, Dalton DL, Lanszki J, et al (2015) Assessment of population structure in hungarian otter populations. J Mammal 96:1275–1283. https://doi.org/10.1093/jmammal/gyv136

McKelvey K (2009) Genetic Errors Associated with Population Estimation Using Non-Invasive Molecular Tagging: Problems and New Solutions. J Wildl Manage 68:439–448. https://doi.org/10.2193/0022-541X(2004)068[0439:GEAWPE]2.0.CO;2

Rico C, Cuesta JA, Drake P, et al (2017) Null alleles are ubiquitous at microsatellite loci in the Wedge Clam (Donax trunculus). PeerJ 5:e3188–e3188. https://doi.org/10.7717/peerj.3188

Sittenthaler M, Schöll EM, Leeb C, et al (2021) Factors influencing genotyping success and genotyping error rate of Eurasian otter (Lutra lutra) faeces collected in temperate Central Europe. Eur J Wildl Res 67:. https://doi.org/10.1007/s10344-020-01444-4

Wahlund S (1928) Zusammensetzung von Populationen und Korrelationserscheinungen vom Standpunkt der Vererbungslehre aus betrachtet. Hereditas 11:65–106. https://doi.org/https://doi.org/10.1111/j.1601-5223.1928.tb02483.x

Waits LP, Luikart G, Taberlet P (2001) Estimating the probability of identity among genotypes in natural populations: cautions and guidelines. Mol Ecol 10:249–256. https://doi.org/https://doi.org/10.1046/j.1365-294X.2001.01185.x
